# The Fate of Dietary Cholesterol in the Kissing Bug *Rhodnius prolixus*

**DOI:** 10.3389/fphys.2021.654565

**Published:** 2021-04-01

**Authors:** Petter F. Entringer, David Majerowicz, Katia C. Gondim

**Affiliations:** ^1^Instituto de Bioquímica Médica Leopoldo de Meis, Universidade Federal do Rio de Janeiro, Rio de Janeiro, Brazil; ^2^Departamento de Biotecnologia Farmacêutica, Faculdade de Farmácia, Universidade Federal do Rio de Janeiro, Rio de Janeiro, Brazil

**Keywords:** cholesterol, intestinal absorption, fat body, ovary, lipid transport

## Abstract

Insects are unable to synthesize cholesterol and depend on the presence of sterols in the diet for cell membrane composition and hormone production. Thus, cholesterol absorption, transport, and metabolism are potential targets for vector and pest control strategies. Here, we investigate the dietary cholesterol absorption and tissue distribution in the kissing bug *Rhodnius prolixus* using radiolabeled cholesterol. Both the anterior and posterior midguts absorbed cholesterol from the ingested blood, although the anterior midgut absorbed more. We also observed esterified cholesterol labeling in the epithelium, indicating that midgut cells can metabolize and store cholesterol. Only a small amount of labeled cholesterol was found in the hemolymph, where it was mainly in the free form and associated with lipophorin (Lp). The fat body transiently accumulated cholesterol, showing a labeled cholesterol peak on the fifth day after the blood meal. The ovaries also incorporated cholesterol, but cumulatively. The insects did not absorb almost half of the ingested labeled cholesterol, and radioactivity was present in the feces. After injection of ^3^H-cholesterol-labeled Lp into females, a half-life of 5.5 ± 0.7 h in the hemolymph was determined. Both the fat body and ovaries incorporated Lp-associated cholesterol, which was inhibited at low temperature, indicating the participation of active cholesterol transport. These results help describe an unexplored part of *R. prolixus* lipid metabolism.

## Introduction

Cholesterol and other sterols are lipid molecules fundamental for cell function, constituting membranes and regulating their fluidity (Yang et al., 2016). Besides, hormones produced from sterols are present in different phyla of the Animal Kingdom (Qu et al., 2015). Mammals absorb cholesterol from their diet, but their primary source of cholesterol is *de novo* synthesis from acetate (Alphonse and Jones, 2016). On the other hand, insects are auxotrophic for dietary sterols, as they can convert sterols into cholesterol (Behmer et al., 1999), but they do not synthesize cholesterol *de novo* due to the lack of essential enzymes in the biosynthetic pathway (Clark and Block, 1959; Li and Jing, 2020). Thus, inhibiting dietary cholesterol absorption may be an effective control action for insect pests and vectors.

Cholesterol metabolism in insects has been studied in a few species, especially in Lepidoptera, Orthoptera, and Diptera. The midgut is the leading site for the absorption of ingested cholesterol in *Manduca sexta* (Jouni et al., 2002b) and in the locusts *Schistocerca gregaria* and *Locusta migratoria* (Upadhyay et al., 2002). It was first shown in *Drosophila melanogaster* that the protein NPC1b (Niemann–Pick type C1b) acts at an early step in sterol incorporation by the insect midgut (Voght et al., 2007). Most insects, differently from vertebrates, have two *NPC1* homolog genes, which encode the plasma membrane protein (NPC1b) or one that is involved in intracellular sterol trafficking (NPC1a) (Jing and Behmer, 2020). Sterol carrier protein-2 (SCP-2) has also been implicated in dietary sterol absorption, although its role is not clear. The gut of the moth *Spodoptera litura* expresses some SCPs in large quantities, and knockdown of these genes reduced the amount of cholesterol present in the hemolymph (Guo et al., 2009). Similarly, the use of pharmacological SCP inhibitors in *M. sexta* reduced cholesterol uptake (Kim and Lan, 2010). Additionally, SCP overexpression in cultured *Aedes aegypti* cells increased cholesterol uptake (Lan and Wessely, 2004). These results indicate that SCPs also take part in cholesterol absorption control.

Intestinal cells esterify part of the absorbed cholesterol (Komnick and Giesa, 1994; Jouni et al., 2002b), although in the hemolymph cholesterol is mostly in the free form and only a very small proportion, if any, is found as cholesterol esters (Chino and Downer, 1982; Komnick and Wachtmann, 1994; Jouni et al., 2002b; Atella et al., 2006; Majerowicz et al., 2013). These results indicate the presence of cholesterol acyltransferase and cholesterol esterase activities in the intestinal cells. In fact, in *D. melanogaster*, the lipase magro, in addition to its luminal triacylglycerol (TAG) lipase activity, also has cholesterol esterase activity inside the midgut cells, being important to maintain cholesterol homeostasis (Sieber and Thummel, 2012). In the gut of the dragonfly *Aeshna cyanea*, a cholesterol esterase activity was also identified, but in that case, it was a luminal activity, probably for digestion of dietary esterified cholesterol (Komnick and Giesa, 1994).

Cholesterol, as other lipids, is transported in the insect hemolymph by lipophorin (Lp) (Chino and Downer, 1982; Dantuma et al., 1997; Jouni et al., 2002a,b, 2003; Yun et al., 2002; Atella et al., 2006; Majerowicz et al., 2013). In *M. sexta* larvae, Lp receives cholesterol from the intestine and delivers it to the fat body (Yun et al., 2002); a long (10 h) half-life was determined after injection of cholesterol-labeled Lp into larval hemolymph (Jouni et al., 2002b).

The presence of either antibodies against the lipid transfer particle (LTP) or suramin in the medium did not affect cholesterol delivery from the midgut to Lp and only partially inhibited cholesterol transfer to and from the fat body (Jouni et al., 2002a; Yun et al., 2002). LTP facilitates diacylglycerol transfer from the midgut to Lp and to the fat body (Canavoso and Wells, 2001; Canavoso et al., 2004b), and suramin affects Lp binding to its receptor (Gondim and Wells, 2000). Thus, based on these and some other results, these authors proposed a predominant role of aqueous diffusion in the cholesterol transfer between Lp and tissues, and that a receptor-mediated process, if present, had a smaller contribution. However, the mechanism involved in cholesterol transport between lipoprotein, other proteins, and cells in insects needs more study.

The fat body stores the hemolymph-derived cholesterol in both free and esterified forms; in larval *A. cyanea* and adult *M. sexta*, cholesterol esters may account for 60–75% of total cholesterol in this organ. On the other hand, in larvae and pupae of *M. sexta*, most of cholesterol found in the fat body is in the free form, unlike the adults of the same species (Komnick and Giesa, 1994; Jouni et al., 2002b). In the triatomines *Dipetalogaster maxima*, *Triatoma infestans*, and *Panstrongylus megistus*, free cholesterol stored in the fat body was consumed during fasting periods, as occurred to other neutral lipids (Canavoso et al., 1998).

Lipophorin also transfers cholesterol to developing oocytes, as shown in *M. sexta* and in the silkworm *Bombyx mori* (Jouni et al., 2002b, 2003). Maternal cholesterol is the major source of embryo sterol content; and, thus, the appropriate ingestion, absorption, and transport of cholesterol to the oocytes are required for successful reproduction (Behmer and Grebenok, 1998; Jing and Behmer, 2020).

*Rhodnius prolixus* is an important vector of Chagas disease in South and Central Americas (de Fuentes-Vicente et al., 2018), and it is a model for insect physiology and biochemistry studies (Nunes-da-Fonseca et al., 2017), including lipid metabolism (Gondim et al., 2018). Our group has already characterized various aspects of *R. prolixus* lipid metabolism, including TAG digestion, absorption, and distribution (Grillo et al., 2007); the interaction of Lp with different organs (Pontes et al., 2002; Grillo et al., 2003; Entringer et al., 2013); and the synthesis and dynamics of TAG in the fat body (Pontes et al., 2008; Alves-Bezerra and Gondim, 2012; Alves-Bezerra et al., 2017) and ovaries (Santos et al., 2011). However, cholesterol metabolism remains a gap in the knowledge of this insect physiology. *R. prolixus* is an exclusive hematophagous hemipteran, feeding on blood during the whole life cycle, nymphs and adults. Although blood contains significant amounts of cholesterol, there is no information about absorption and utilization of this lipid in any triatomine species. Thus, here, we used radiolabeled cholesterol to investigate the fate of dietary cholesterol ingested by *R. prolixus* during the blood meal.

## Materials and Methods

### Insects

The experimental insects were adult mated females of *R. prolixus* taken from a colony kept at 28 ± 2°C, 75–85% relative humidity, and 12-h/12-h light and dark cycles. The insects were fed on the outer ears of live rabbits at 3-week intervals. All animal care and experimental protocols were conducted following the guidelines of the institutional animal care and use committee (Committee for Evaluation of Animal Use for Research from the Universidade Federal do Rio de Janeiro, CEUA-UFRJ), process number 01200.001568/2013-87, order number 149/19, and the NIH Guide for the Care and Use of Laboratory Animals (ISBN 0-309-05377-3).

### Absorption and Distribution of ^3^H-Cholesterol Added to a Blood Meal

Adult females of *R. prolixus* were fed in an artificial feeder (Garcia et al., 1975) on whole rabbit blood supplemented with ^3^H-cholesterol, 10^6^ disintegrations per minute (DPM)/ml of blood ([1,2-^3^H(N)]-cholesterol, specific activity 51 Ci/mmol; PerkinElmer, Boston, MA, United States; 3 ng of ^3^H-cholesterol/ml of blood). About 1 h after blood meal, insects were individually housed at 28°C. On the first, third, fifth, seventh, and 10th days after the ^3^H-cholesterol-enriched blood meal, hemolymph was individually collected in the presence of phenylthiourea. Insects were dissected; and anterior midgut, posterior midgut, ovaries, and fat bodies were collected. The midgut tissue was separated from the luminal content, by individually placing the anterior and posterior midguts in 500 μl of cold water. The obtained organs were abundantly washed in phosphate-buffered saline (PBS; 10 mM of sodium phosphate, 0.15 M of NaCl, pH 7.4) and homogenized in 200 μl of the same buffer. The hemolymph, homogenates, and luminal contents were frozen for posterior analysis. When present (usually on the seventh and 10th days), total feces from the dissected insects were collected by washing the vial bottom with 500 μl of PBS.

### Lipid Extraction and Cholesterol Analysis

Organ homogenates, hemolymph, and midgut luminal contents were subjected to lipid extraction (Blight and Dyer, 1959). The organic phases containing the lipids were dried under a stream of nitrogen, and lipids were resuspended in 30 μl of chloroform and analyzed by high-performance thin-layer chromatography (HPTLC) on Silica gel 60 plates (Merck, Darmstadt, Germany) developed in hexane:ethyl ether:acetic acid (60:40:1) as solvent (Kawooya and Law, 1988). The following commercial lipid standards were used: cholesterol, cholesteryl ester, free fatty acid, monoacylglycerol, diacylglycerol, and TAG (Sigma–Aldrich, St. Louis, MO, United States). Plates were stained with iodine vapor, the spots corresponding to free and esterified cholesterol were scraped, and the lipids were eluted three times from the silica with chloroform:methanol:water (1:2:1). The lipids were extracted from the supernatants in chloroform, and the radioactivity was determined by scintillation counting.

### Identification of the Hemolymphatic Cholesterol Carrier

In order to follow cholesterol association with hemolymphatic lipoproteins, 40 adult females were fed on ^3^H-cholesterol-supplemented blood as described in Section “Absorption and Distribution of ^3^H-Cholesterol Added to a Blood Meal,” and 6–7 days later, hemolymph was collected in the presence of 0.15 M of NaCl, 5 mM of ethylenediamine tetraacetic acid (EDTA), and protease inhibitor cocktail (Sigma–Aldrich). The hemolymph pool was centrifuged at 12,000 × *g* for 5 min at 4°C to remove cells. KBr was added to the supernatant to a final concentration of 44.5% in PBS, and this solution (10 ml) was overlaid with 10 ml of 11% KBr in PBS (Pontes et al., 2002). This material was centrifuged at 159,000 × *g* for 20 h at 4°C, in a Beckman Coulter (Fullerton, CA, United States) 70 Ti rotor. The gradient was fractionated from the top to the bottom; and the radioactivity associated with 10 μl of each fraction was determined by scintillation counting. To remove the KBr from the gradient fractions, Sephadex G-50 (Sigma–Aldrich) spin columns (Penefsky, 1977) equilibrated with PBS were used. Fractions were analyzed by sodium dodecyl sulfate–polyacrylamide gel electrophoresis (SDS-PAGE) (6–22%) (Laemmli, 1970) and stained with Coomassie blue.

### *In vitro* Lipophorin Labeling With ^3^H-Cholesterol

To obtain a purified ^3^H-cholesterol-labeled Lp, an *in vitro* labeling protocol was adapted from one described for human low-density lipoprotein (LDL) (Coppens et al., 1995). Hemolymph was collected from adult females 4–5 days after a blood meal, in the presence of 0.15 M of NaCl, 5 mM of EDTA, and protease inhibitor cocktail (Sigma–Aldrich). After centrifugation to remove cells, 100 μCi of ^3^H-cholesterol (0.7 μg) was added to the supernatant (5 ml). This material was incubated for 16 h at 28°C. Then, the ^3^H-cholesterol-containing hemolymph was subjected to a KBr ultracentrifugation gradient, as described in Section “Identification of the Hemolymphatic Cholesterol Carrier.” The gradient was fractionated from top to bottom. The radioactivity present in 10 μl of each fraction was determined by scintillation counting. ^3^H-Cholesterol-Lp was localized in fractions 1–4, which were pooled, extensively dialyzed against PBS, concentrated using Centricon^®^ (Millipore, Bedford, MA, United States), and stored under liquid nitrogen until use.

To confirm the association of ^3^H-cholesterol with Lp, an aliquot of pooled fractions 1–4 was subjected to a second KBr ultracentrifugation gradient, where KBr concentration was adjusted to 44.5% in PBS (5 ml), and it was overlaid with 5 ml of PBS (0–44.5% KBr gradient). This material was centrifuged as described in Section “Identification of the Hemolymphatic Cholesterol Carrier,” in a 70.1 Ti rotor. The gradient was fractionated from the top in 500-μl fractions; the radioactivity and absorbance at 280 nm were determined in each one. The density of fractions was determined by the refractive index of KBr at 25°C. To identify the fractions where the ^3^H-cholesterol alone (non-associated with proteins) would localize, 100 μCi of ^3^H-cholesterol was mixed with nonradioactive cholesterol (20 μg), and the mixture was subjected to an identical KBr gradient (0–44.5%). The gradient was fractionated in the same way, and the radioactivity present in each fraction was determined by scintillation counting.

### ^3^H-Cholesterol-Lipophorin Injection

On the third day after a blood meal, *R. prolixus* adult females were injected with 5 μl of ^3^H-cholesterol-Lp (∼10^5^ DPM) into the hemocoel, using a 10-μl syringe (Hamilton, Reno, NV, United States), and kept at 28 or 4°C. At different times, 5 μl of hemolymph, ovaries, and fat bodies were collected. After being washed, the organs were homogenized in 0.15 M of NaCl, and radioactivity associated with the samples was determined by scintillation counting. Results obtained for hemolymph were also used for the determination of ^3^H-cholesterol-Lp half-life in adult female hemolymph, as described elsewhere (Soulages and Wells, 1994a).

## Results

**FIGURE 1:**
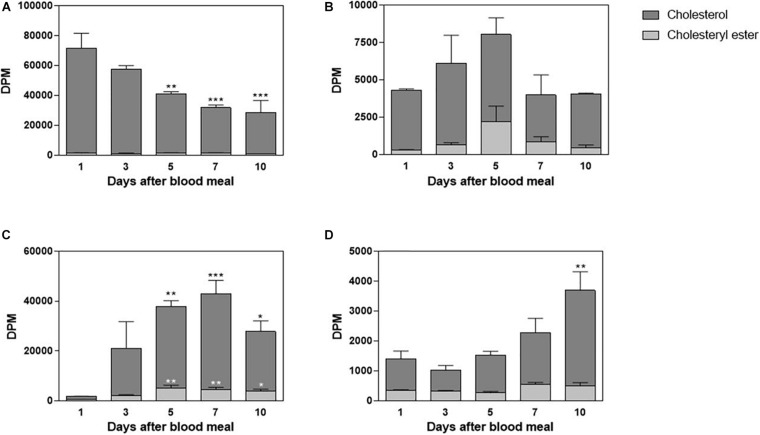
Cholesterol absorption by the midgut. *Rhodnius prolixus* adult females were fed on ^3^H-cholesterol-enriched blood and dissected on different days after the blood meal. Lipids from anterior midgut content **(A)** and tissue **(B)**, as well as from posterior midgut content **(C)** and tissue **(D)**, were extracted and analyzed by HPTLC. Radioactivity associated with free cholesterol (dark bars) and esterified cholesterol (light bars) was quantified and expressed as DPM/content or tissue ± SEM for three to four determinations. (*), (**), and (***): significantly different from day 1 after analysis by one-way ANOVA followed by Dunnett’s posttest, *p* < 0.05, *p* < 0.01, and *p* < 0.001, respectively. HPTLC, high-performance thin-layer chromatography; DPM, disintegrations per minute.

*Rhodnius prolixus* feeds solely on blood, and although cholesterol is present in blood in considerable amounts, there is no information about its midgut traffic and absorption during digestion. Hemipteran midgut is composed of the anterior midgut (or stomach), where the blood meal is stored and digestion initiates, and the posterior midgut (or intestine), where blood components are mostly digested and absorbed (Billingsley, 1990). In this way, in order to investigate cholesterol metabolism in this triatomine, adult females were fed on blood enriched with labeled cholesterol, ^3^H-cholesterol; and radioactivity was chased in midgut tissue and luminal contents, on the days after the blood meal. The amount of ^3^H-cholesterol in the luminal contents of the anterior midgut progressively decreased; and by the fifth day after feeding, it was significantly lower than on the first day. A very small amount of radiolabeled esterified cholesterol could be detected (Figure 1A). The anterior midgut cells absorbed cholesterol from the diet, although this lipid did not seem to accumulate in the organ. A significant amount of labeled esterified cholesterol was also detected in these intestinal cells (Figure 1B). In the posterior midgut luminal content, the amount of labeled cholesterol increased as ingested blood moved from the anterior to the posterior midgut, and it reached higher levels on the fifth day after feeding. Significant levels of labeled esterified cholesterol also appeared during this period (Figure 1C). The cells of the posterior midgut also absorbed blood cholesterol and the lipid accumulated in the free form; its amount was higher on the 10th day after feeding, whereas labeled esterified cholesterol amounts remained low and constant (Figure 1D).

**FIGURE 2:**
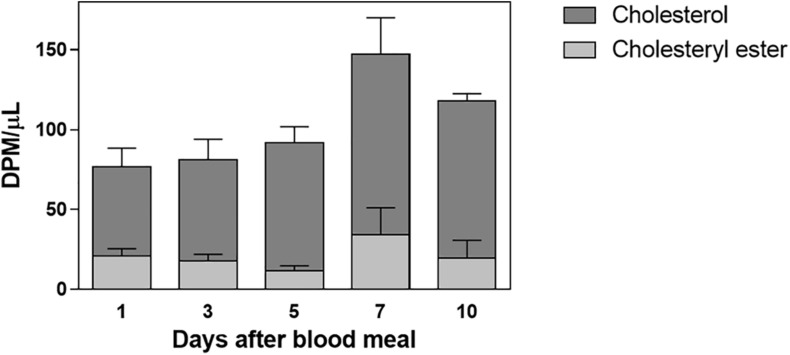
Labeled cholesterol is found in the hemolymph after a blood meal. *Rhodnius prolixus* adult females were fed on ^3^H-cholesterol-enriched blood, and on different days after the blood meal hemolymph was collected. Total lipids were extracted from the hemolymph and were analyzed by HPTLC. The radioactivity associated with free cholesterol (dark bars) and esterified cholesterol (light bars) was quantified and expressed as DPM/μl ± SEM for two to three determinations. *p* > 0.05 by one-way ANOVA. HPTLC, high-performance thin-layer chromatography; DPM, disintegrations per minute.

**FIGURE 3:**
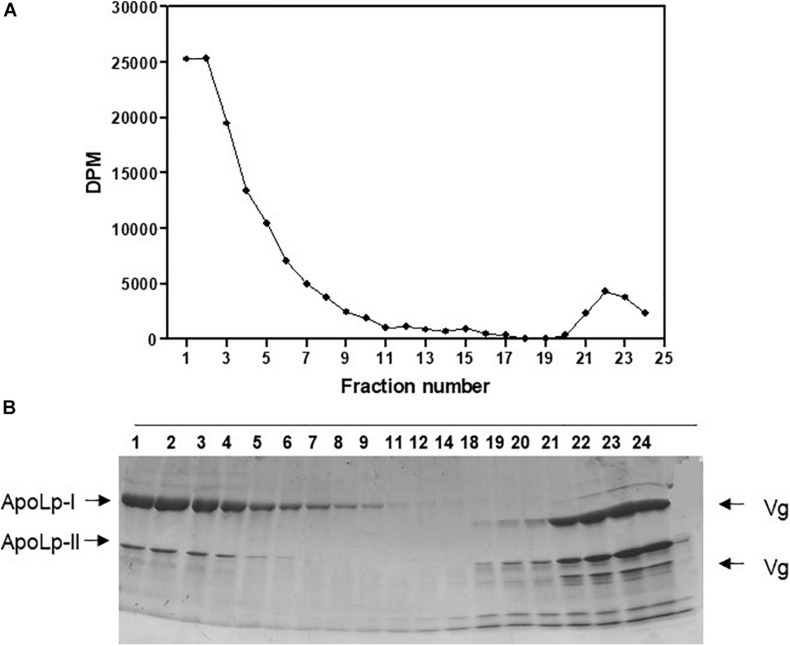
Absorbed cholesterol associates with hemolymphatic lipophorin. *Rhodnius prolixus* adult females were fed on ^3^H-cholesterol-enriched blood and on the sixth day after the blood meal hemolymph was collected and subjected to a density gradient ultracentrifugation. **(A)** Radioactivity present in gradient fractions was quantified and expressed as DPM/fraction. **(B)** Fractions were analyzed by SDS-PAGE (6–22%). Results shown are representative of four experiments. ApoLp-I, apolipophorin-I; ApoLp-II, apolipophorin-II; Vg, vitellogenin; DPM, disintegrations per minute; SDS-PAGE, sodium dodecyl sulfate–polyacrylamide gel electrophoresis.

Labeled free and esterified cholesterol reached the hemolymph on the first day after feeding. However, hemolymphatic radioactivity levels always remained low and without significant changes; again, most of the labeling was found in free cholesterol (Figure 2). To identify the proteins that bound circulating cholesterol, the hemolymph was subjected to a KBr density gradient, and as expected, cholesterol was mostly associated with Lp, on the top of the gradient (Figure 3).

**FIGURE 4:**
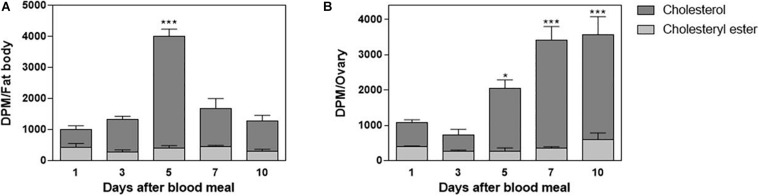
The fat body and ovary incorporate cholesterol from the blood meal. *Rhodnius prolixus* adult females were fed on ^3^H-cholesterol-enriched blood and dissected on different days after the blood meal. Total lipids from the fat body **(A)** or ovaries **(B)** were extracted and were analyzed by HPTLC. Radioactivity associated with free cholesterol (dark bars) and esterified cholesterol (light bars) was quantified and expressed as DPM/organ ± SEM for three to four determinations. (*) and (***): significantly different from 1 day after analysis by one-way ANOVA followed by Dunnett’s posttest, *p* < 0.05 and *p* < 0.001, respectively. HPTLC, high-performance thin-layer chromatography; DPM, disintegrations per minute.

In order to understand cholesterol distribution by Lp after the lipoprotein received it from the midgut, other organs were dissected from the ^3^H-cholesterol-fed females on the days after the blood meal. Both the fat body and ovaries showed incorporated dietary cholesterol on the first day after feeding. In the fat body, this cholesterol accumulation was dynamic, with a peak of accumulation on the fifth day after feeding (Figure 4A). In the ovaries, cholesterol accumulation progressively increased, in accordance with ovarian growth during vitellogenesis, with higher levels of labeled lipid observed from the fifth to 10th days after feeding (Figure 4B). The esterified cholesterol was present in lower and constant proportions throughout the analyzed period in both organs.

**FIGURE 5:**
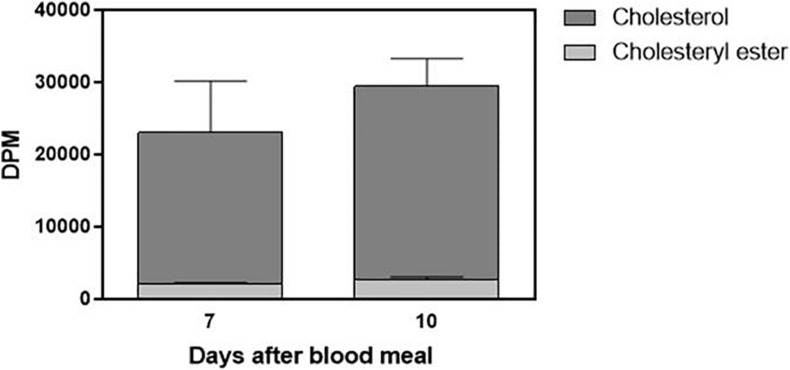
Part of ingested cholesterol is excreted in feces. Feces (total amount produced after feeding) from *Rhodnius prolixus* adult females fed on ^3^H-cholesterol-enriched blood were collected on the seventh and 10th days after the blood meal. Lipids were extracted from the feces and were analyzed by HPTLC. Radioactivity associated with free cholesterol (dark bars) and esterified cholesterol (light bars) was quantified and expressed as DPM/total feces ± SEM from three to four determinations. *p* > 0.05 by the Mann–Whitney test. HPTLC, high-performance thin-layer chromatography; DPM, disintegrations per minute.

Cholesterol absorption from the diet was not complete, and the insect feces contained a significant amount of labeled cholesterol (Figure 5). The presence of a small amount of the esterified lipid is noteworthy as well. Digestion in *R. prolixus* is very slow, and feces were present on the seventh and 10th days after feeding. Sometimes, there were feces on the fifth day, but this was not a general pattern, so we did not analyze fecal samples from the insects dissected at this day.

**FIGURE 6:**
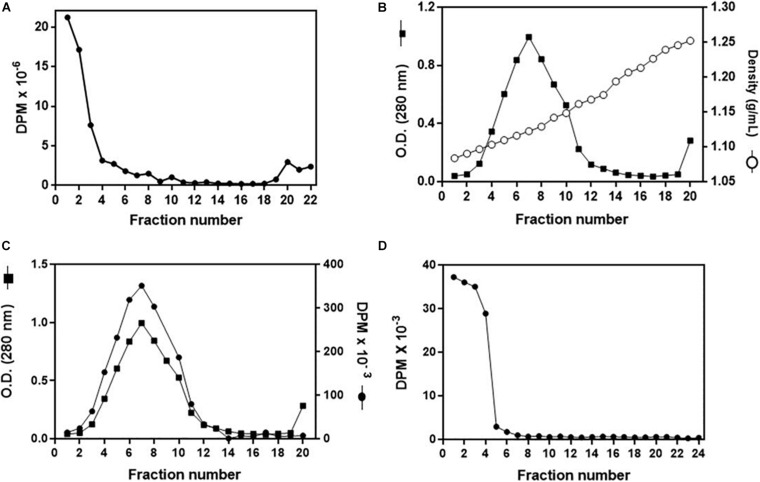
Analysis of *in vitro* ^3^H-cholesterol incorporation by lipophorin (Lp). **(A)** Non-radioactive hemolymph was incubated with ^3^H-cholesterol for 16 h, and the mixture was analyzed by a KBr gradient ultracentrifugation (11–44.5% KBr). Radioactivity present in the gradient fractions was determined. **(B)** Fractions 1–3 from the gradient shown in **(A)** were pooled, and the sample was subjected to a second density gradient ultracentrifugation (0–44.5% KBr). The O.D. (280 nm) and density of each gradient fraction were determined. In **(C)**, values of O.D. are the same as in **(B)**, and the radioactivity present in each fraction was also determined. **(D)** ^3^H-cholesterol (100 μCi + 20 μg non-radioactive cholesterol) was subjected to the same KBr gradient ultracentrifugation as in **(B,C)** in the absence of Lp, and the radioactivity present in the gradient fractions was determined. Results shown are representative of three experiments.

To investigate in more detail the cholesterol uptake by the organs, and to confirm that it was delivered by Lp, we established a protocol for the *in vitro* association of ^3^H-cholesterol with Lp. After incubation of the labeled lipid with non-radioactive hemolymph and analysis of the mixture by a KBr gradient, most of the radioactivity was present in the less dense fractions at the gradient top, where Lp was also located (Figure 6A). To verify whether the ^3^H-cholesterol was associated with the lipoprotein, and not only floating on the gradient top, the four fractions from the top were pooled and analyzed on a second KBr gradient ultracentrifugation, where Lp located below the top, in accordance with its density as a high-density Lp (Coelho et al., 1997). The labeled cholesterol appeared associated with Lp, in the expected density range (Figures 6B,C). It is important to note that when ^3^H-cholesterol was subjected to this same KBr gradient condition in the absence of Lp, it located on the lower-density fractions on the gradient top (Figure 6D).

**FIGURE 7:**
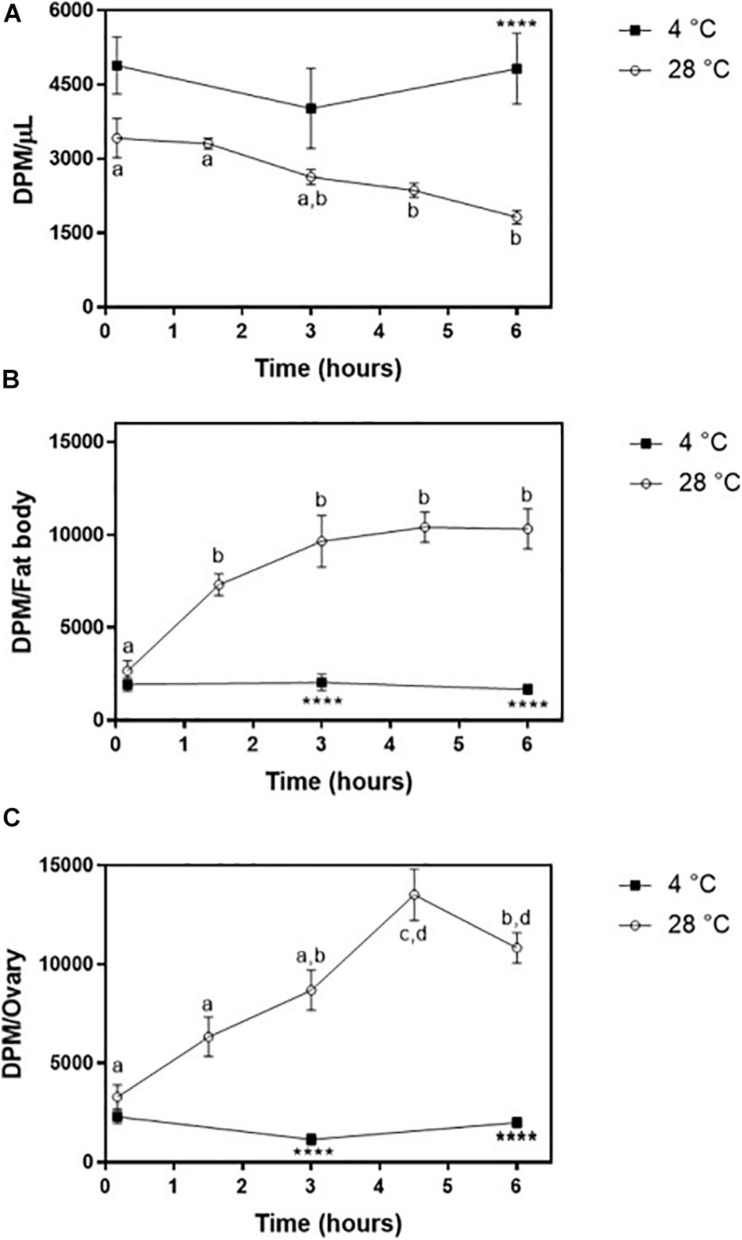
Lipophorin transport of ^3^H-cholesterol to the fat body and ovaries. *Rhodnius prolixus* adult females were injected with ^3^H-cholesterol-Lp on the third day after the blood meal and maintained to 28°C (○) or 4°C **(◼)**. **(A)** At the indicated times, hemolymph was collected, and radioactivity was quantified and expressed as DPM/μl ± SEM for six to 12 determinations. The same insects were dissected, and fat body **(B)** and ovaries **(C)** were isolated. After homogenization, radioactivity in the organs was determined and expressed as DPM/organ ± SEM for 7–12 determinations. Different letters indicate statistical difference between the values of insects at 28°C by one-way ANOVA followed by Tukey’s posttest. (****): significant difference between 4 and 28°C by two-way ANOVA followed by Sidak’s multiple comparisons posttest, *p* < 0.0001. DPM, disintegrations per minute.

The obtained purified radiolabeled Lp (fractions 1–4 of the 11–44.5% KBr gradient) was then injected into adult females on the third day after feeding. At this time point, the fat body was shown to synthesize and accumulate lipid stores very actively, and TAG contents are high; ovaries are rapidly growing, as oocytes are developing and accumulating yolk (Pontes et al., 2008; Santos et al., 2011). ^3^H-Cholesterol was progressively cleared from the hemolymph, with a significant decrease after 3 h (Figure 7A) and a determined half-life of 5.5 ± 0.7 h. The fat body incorporated cholesterol rapidly, reaching maximal levels after 90 min (Figure 7B). Lp also transferred cholesterol to the ovaries, which reached maximal accumulation of radioactivity at 4 h 30 min after injection (Figure 7C). In both organs, radioactivity association was very fast and detected at the first time point (10 min), probably due to the binding of labeled Lp to cell surface, which is a very fast event (Gondim et al., 1989; Pontes et al., 2002; Entringer et al., 2013). As previously observed for diacylglycerol, phospholipids, and fatty acids (Gondim et al., 1989; Pontes et al., 2008; Santos et al., 2011), keeping the insects at 4°C inhibited cholesterol transfer from Lp to the tissues.

## Discussion

As in other insects already studied (Komnick and Giesa, 1994; Jouni et al., 2002b; Upadhyay et al., 2002), *R. prolixus* absorbed cholesterol from the diet, and this lipid accumulated in the fat body and ovaries, being associated with Lp in the hemolymph.

Our results showed that the anterior midgut absorbs cholesterol, as labeling could be found (in both free and esterified forms) in the tissue of this midgut portion. In hemipterans, the anterior midgut stores and concentrates the ingested blood and serves as a storage site for lipids (Billingsley, 1990). Generally, in triatomines, lipids are processed and absorbed in the posterior midgut (Rimoldi et al., 1985; Grillo et al., 2007), although in *P. megistus*, significant TAG digestion occurs in the anterior midgut (Canavoso et al., 2004a). Results from our group showed that the anterior midgut can also absorb free fatty acids present in blood (Majerowicz, unpublished results). These results indicate that the anterior midgut may have a more critical role in lipid metabolism than initially thought.

The presence of labeled esterified cholesterol in the midgut lumen is also an interesting finding. A simple hypothesis to explain this finding would be the presence of lecithin:cholesterol acyltransferase activity in the blood. This enzyme catalyzes the synthesis of esterified cholesterol and lysophosphatidylcholine from cholesterol and phosphatidylcholine (Ossoli et al., 2016), and it is present in the rabbit blood used for insect feeding (Carlucci et al., 2012). A recent proteomic analysis of *R. prolixus* midgut showed that enzymes involved with lipid utilization are highly expressed in this organ, confirming that it is very active in terms of lipid metabolism (Gumiel et al., 2020). Thus, an alternative explanation may be the excretion of esterified cholesterol synthesized by the intestinal cells. The intestines of *M. sexta* and *A. cyanea* esterify incorporated free cholesterol (Komnick and Giesa, 1994; Jouni et al., 2002b), and here, we showed that this is true for *R. prolixus* as well. In this way, the intestinal cells could excrete this esterified cholesterol back to the intestinal lumen. In mammals, two ATP-binding cassette transporters (ABCG5 and ABCG8) are responsible for this excretion activity (Patel et al., 2018). Orthologs of these genes are present in the genomes of moths, mosquitoes, and leafhoppers (Hull et al., 2014; Guo et al., 2015; Lu et al., 2016), but there is no information about their expression profile or biological activity. Future experiments of functional genomics in *R. prolixus* might help to investigate this hypothesis.

The observed increase in ^3^H-cholesterol amount in posterior midgut tissue is possibly due to the incorporation of this lipid into newly synthesized microvillar and perimicrovillar membranes, where cholesterol seems to be abundant and important to maintain their spatial architecture (Albuquerque-Cunha et al., 2009).

Lipophorin transports cholesterol in the hemolymph, as expected, and the observed labeling ratio between free and esterified cholesterol reflects the ratio already described for *R. prolixus* Lp, which contains much smaller amounts of esterified cholesterol than of the free form (1.56 and 10.0% of total lipids, respectively) (Majerowicz et al., 2013). In the same way, in similar experiments where radioactive cholesterol was fed to the larvae of *M. sexta* and *A. cyanea*, in both cases, labeled cholesterol was found in the hemolymph in the free form, exclusively or almost. These results are in accordance with the fact that, unlike vertebrate lipoproteins, Lps of different insect species show a complete absence, or tiny amounts, of cholesterol esters (Soulages and Wells, 1994b; Majerowicz et al., 2013). The observed amount of labeled cholesterol in the hemolymph was always low despite its low decay rate in the hemolymph, as determined after injection of ^3^H-cholesterol-labeled Lp.

Dietary cholesterol was incorporated by both the fat body and ovaries, but with different dynamics. In the fat body, the radioactivity levels peaked on the fifth day after feeding and on the seventh day had already returned to the initial levels. This profile indicated high uptake and outflow rates of cholesterol from the fat body. Accordingly, Lp binding to its receptors in the fat body membrane is maximal on the fifth day after feeding, and the uptake of fatty acids from the hemolymph by the fat body is also high around this time (Pontes et al., 2008). On the other hand, the delivery of phospholipids and diacylglycerol from the fat body to Lp is highest around the 10th day after feeding (Coelho et al., 1997). Since Lp also carries cholesterol, the dynamics of this transport can possibly follow a similar pattern as for other lipids. Interestingly, in other triatomines, the relative amounts of cholesterol in the fat body were constant during a long period after feeding, although the total lipid content varied (Canavoso et al., 1998), indicating that the cholesterol stock is dynamic and affected by the insect physiological condition. Most of fat body-incorporated cholesterol was in the free form, as observed for *M. sexta* larvae and pupae (Jouni et al., 2002b).

In the ovaries, cholesterol started to accumulate in the first day after feeding, and total incorporated radioactivity significantly increased at day 5. Differently from the fat body, cholesterol remained stored in the ovaries, as oocytes developed during this time. This is in accordance with the maternal origin of most of sterol content in embryos, accumulated during oogenesis (Jing and Behmer, 2020). The ovaries capture fatty acids, diacylglycerol, and phospholipids from Lp for oocyte growth during vitellogenesis (Gondim et al., 1989; Santos et al., 2011), and cholesterol is possibly simultaneously taken up. Similarly to the fat body, most of ^3^H-cholesterol found in the ovaries was in the free form, unlike the observed in *M. sexta* oocytes, where the free and esterified forms were almost equally distributed (Jouni et al., 2002b). In *D. melanogaster*, high levels of esterified cholesterol were also found in early embryos (Guan et al., 2013).

Interestingly, the females did not absorb a great part of the labeled cholesterol added to the blood meal, which ended up excreted in feces. As insects are auxotrophic for cholesterol, we would expect high absorption efficiency. On the other hand, it is possible that, as the blood is very rich in cholesterol, it is more than enough to attend to the insects’ demands, so that a significant part of the ingested cholesterol is not absorbed, with no impact on the insect physiology.

Both fat body and ovaries incorporated cholesterol associated with Lp with similar efficiency. However, incubation of insects at 4°C blocked this uptake, as also observed for other lipid classes, as fatty acids, diacylglycerol, and phospholipids in *R. prolixus*, as well as for cholesterol in *M. sexta* (Gondim et al., 1989; Jouni et al., 2002a; Pontes et al., 2008; Santos et al., 2011), indicating that cholesterol incorporation by the organs depends on active metabolism and/or membrane properties. At 4°C, probably only Lp binding to cell surface occurred.

In summary, we showed here that *R. prolixus* adult females absorb cholesterol from the blood meal, that it is transferred to circulating Lp, and that it transports and delivers this lipid to the fat body and ovaries, where it is stored mostly as free cholesterol.

## Data Availability Statement

The raw data supporting the conclusions of this article will be made available by the authors, without undue reservation.

## Ethics Statement

The animal study was reviewed and approved by the Committee for Evaluation of Animal Use for Research from the Universidade Federal do Rio de Janeiro, CEUA-UFRJ.

## Author Contributions

PE designed and conducted the experiments, analyzed the results, and revised the manuscript. DM analyzed the results and wrote the manuscript. KG designed the experiments and wrote the manuscript. All authors contributed to the article and approved the submitted version.

## Conflict of Interest

The authors declare that the research was conducted in the absence of any commercial or financial relationships that could be construed as a potential conflict of interest.

## Abbreviations

ApoLp-I: apolipophorin-I
ApoLp-II: apolipophorin-II
EDTA: ethylenediamine tetraacetic acid
HPTLC: high-performance thin-layer chromatography
Lp: lipophorin
LTP: lipid transfer particle
NPC: Niemann–Pick type C protein
PBS: phosphate-buffered saline
SCP: sterol carrier protein
TAG: triacylglycerol
Vg: vitellogenin.
